# antibacTR: dynamic antibacterial-drug-target ranking integrating comparative genomics, structural analysis and experimental annotation

**DOI:** 10.1186/1471-2164-15-36

**Published:** 2014-01-17

**Authors:** Alejandro Panjkovich, Isidre Gibert, Xavier Daura

**Affiliations:** Institute of Biotechnology and Biomedicine (IBB), Universitat Autònoma de Barcelona (UAB), 08193 Bellaterra, Spain; Departament de Genètica i de Microbiologia, UAB, 08193 Bellaterra, Spain; Catalan Institution for Research and Advanced Studies (ICREA), 08010 Barcelona, Spain

**Keywords:** Antibacterial-drug target, Gram-negative bacteria, Target druggability

## Abstract

**Background:**

Development of novel antibacterial drugs is both an urgent healthcare necessity and a partially neglected field. The last decades have seen a substantial decrease in the discovery of novel antibiotics, which combined with the recent thrive of multi-drug-resistant pathogens have generated a scenario of general concern. The procedures involved in the discovery and development of novel antibiotics are economically challenging, time consuming and lack any warranty of success. Furthermore, the return-on-investment for an antibacterial drug is usually marginal when compared to other therapeutics, which in part explains the decrease of private investment.

**Results:**

In this work we present antibacTR, a computational pipeline designed to aid researchers in the selection of potential drug targets, one of the initial steps in antibacterial-drug discovery. The approach was designed and implemented as part of two publicly funded initiatives aimed at discovering novel antibacterial targets, mechanisms and drugs for a priority list of Gram-negative pathogens: *Acinetobacter baumannii*, *Escherichia coli*, *Helicobacter pylori*, *Pseudomonas aeruginosa* and *Stenotrophomonas maltophilia*. However, at present this list has been extended to cover a total of 74 fully sequenced Gram-negative pathogens. antibacTR is based on sequence comparisons and queries to multiple databases (*e.g.* gene essentiality, virulence factors) to rank proteins according to their potential as antibacterial targets. The dynamic ranking of potential drug targets can easily be executed, customized and accessed by the user through a web interface which also integrates computational analyses performed in-house and visualizable on-site. These include three-dimensional modeling of protein structures and prediction of active sites among other functionally relevant ligand-binding sites.

**Conclusions:**

Given its versatility and ease-of-use at integrating both experimental annotation and computational analyses, antibacTR may effectively assist microbiologists, medicinal-chemists and other researchers working in the field of antibacterial drug-discovery. The public web-interface for antibacTR is available at ‘http://bioinf.uab.cat/antibactr’.

## Background

Since their initial discovery and application during the early 20th century, antibiotics have been playing a key role in public health worldwide. These ‘miracle drugs’ have contributed significantly to the increase in life expectancy since the end of World War II. Besides curing infections, they also prevent amputations and blindness and are involved in multiple healthcare procedures such as joint-replacement, surgery, new cancer treatments, etc [1]. However, after peaking during the 1960’s, the discovery of new antibiotics has fallen off dramatically. The present scarcity of novel antibiotics becomes a major health concern in light of the remarkable ability of bacteria to rapidly evolve resistance mechanisms which erode the therapeutic effect of known antibiotics [2]. Nowadays multi-drug-resistant bacterial infections are increasing in both developing and developed countries and in both community and nosocomial settings [3]. It has been reported that a number of pathogens, including *Staphylococcus aureus*, *Mycobacterium tuberculosis*, *Pseudomonas aeruginosa*, *Acinetobacter baumannii* and some Enterobacteriaceae have developed resistance to a wide range of antimicrobial agents at an alarming rise, with some strains becoming truly pan-resistant [4, 5]. However, pharmaceutical companies have not been investing in the development of new antibacterial drugs with corresponding efforts, mainly due to economic criteria that favour other therapeutic areas with better return-on-investment ratios [6, 7]. The few antibacterial agents that have been launched during the last decade (*e.g.* linezolid, daptomycin) have a good activity against Gram-positive bacteria such as methicillin-resistant *S. aureus* (MRSA) and vancomycin-resistant enterococci [8]. However, cases of resistance for these new Gram-positive antibiotics have been reported recently as well [9].

The situation is worse for Gram-negative bacteria, such as *P. aeruginosa* and *A. baumannii*, which are common among nosocomial infections [10] and for which no new antibiotics have reached advanced stages of development [1]. In addition, with the increase in the prevalence of extended spectrum *β*-lactamase (ESBL)-producing Enterobacteriaceae, the use of carbapenems, a potential alternative to treat infections caused by these microorganisms, is leading to the emergence of multi-drug-resistant Enterobacteriaceae including resistance to carbapenems [11].

This scenario emphasizes the relevance of initiatives focused on the discovery of novel targets and antibacterials for combating Gram-negative pathogens. Here, we describe a tool (antibacTR: antibacterial Target Ranking) to support the initial stages of selection of potential antibacterial-drug targets, developed within the context of two such initiatives. antibacTR integrates a database with a pipeline that ranks and filters proteins according to a set of criteria commonly associated to antibacterial targets. The approach is based on protein sequence comparisons, for which we developed an unbiased measure described in the Methods Section.

The interface used to interrogate the database and access the results has the form of a web-based tool, which has been developed following the suggestions of the experimentalists involved in the two target-discovery initiatives. It includes access to thousands of three-dimensional protein-structure models that can be visualized and downloaded for further analysis through the web-interface. To further exploit the structural models, we incorporated a predictive approach that evaluates putative ligand-binding pockets in terms of their potential to affect protein function upon ligand binding [12, 13]. Links to DrugBank [14] and the Virulence Factors DataBase (VFDB) [15], as well as predictions of active-site residues are provided as well. It should be noted that the final aim of the tool is both to rank proteins according to the chosen set of criteria (with weights defined by the user) and to provide for each protein in the ranked list information that could be relevant to antibacterial-drug-target selection. Clearly, the drug-target property is the result of a complex combination of a variable number of non-universal factors, some of which having opposite sign for different types of targets or mechanisms. Thus, this is a tool to support target discovery efforts, not a target-prediction tool. In other words, it will not spare the user from scanning and evaluating a large number of proteins, it will simply provide him/her with additional means to do it.

All measurements and predictions are pre-computed, which allows the application to return full rankings and links to the relevant information within seconds. This characteristic distinguishes our tool from similar ones such as the UniDrug-Target (UDT) database, which can be used to perform comparative analyses online with computation at time of request [16]. Besides execution speed, our approach differs from UDT and related ones such as the Prokaryotic-genome Analysis Tool (PGAT) [17] in its focus. While these tools succeed at providing comparative-analysis means that can be used through a web-interface, our dynamic approach focuses on speed, ease of use and an integrative solution that allows the user to quickly scan putative antibacterial targets and relevant information such as three-dimensional structural models and other predictions, while the comparative analysis is just one of the underlying features.

Other researchers have focused on extensive, manually curated analyses of a single organism and strain, like Shanmugham and Pan [18] on *Mycobacterium abscessus* ATCC 19977. Instead, we have chosen to sacrifice part of the depth of our analysis to cover a much larger selection of pathogens. The methodology presented here was originally developed for two specific projects and their target bacteria, including three of the big four Gram-negative pathogens in relation to systemic infections, *i.e.Escherichia coli*, *Klebsiella pneumoniae*, *Acinetobacter baumannii* and *Pseudomonas aeruginosa*[4]. However, we have now expanded the coverage to include 74 Gram-negative pathogens, which should make the system useful to many more researchers in the field. Through this article, we describe the approach and make the web-based tool publicly available.

## Results

### Computational target-ranking pipeline

Typically, one of the first steps in a target-discovery project is to readily select, among thousands of proteins composing the pathogens’ proteomes, those with the highest chance of becoming useful therapeutic targets. Following the lines defined by previous studies [19], we developed an algorithm to score and rank potential drug targets in pathogenic organisms by evaluating a modular set of criteria that are commonplace in antimicrobial-development efforts [7]: 1) the presence of the protein in different pathogens, 2) evolutionary conservation, 3) essentiality, 4) presence of isoforms and paralogs in the proteome, 5) similarity to human proteins. We implemented a set of five weighted scores that cover these criteria and defined a scoring function combining them.

The first two concepts were incorporated as two independent scores, measuring the conservation of the protein among Gram-negative organisms and among different strains of the same species, respectively. Conservation among strains is a basic requirement for target consideration. Conservation among Gram-negative species is highly desirable as it enables the development of broad-spectrum solutions and increases economic viability. In addition, well conserved targets will presumably have low tolerance to mutations, decreasing the chance of resistance to emerge by this type of mechanism.

Essential proteins, which inhibition compromises bacterial viability, are potential antibacterial targets by definition. We implemented a binary score by marking genes known to be essential from previous experimental work [20].

The remaining two scores are given negative weights. If the protein under consideration has isoforms and/or paralogs the pathogen may readily develop resistance by functional substitution, and the effect of the antibacterial may be also reduced by competitive binding to non-essential forms. We considered similarity to human proteins negative as well, since close human homologs to the target may interact with the drug, giving rise to unwanted side-effects.

The scoring and ranking scheme, partially following the work of White and Kell [19] provides an advantage when compared to static selection or filtering approaches [16, 21]. In our case, if further experimental analysis reveals that a given protein is not suitable as a drug target, work can continue with the next protein in the ranking. Moreover, it would be straightforward to incorporate new criteria into the ranking scheme if needed.

**Figure 1:**
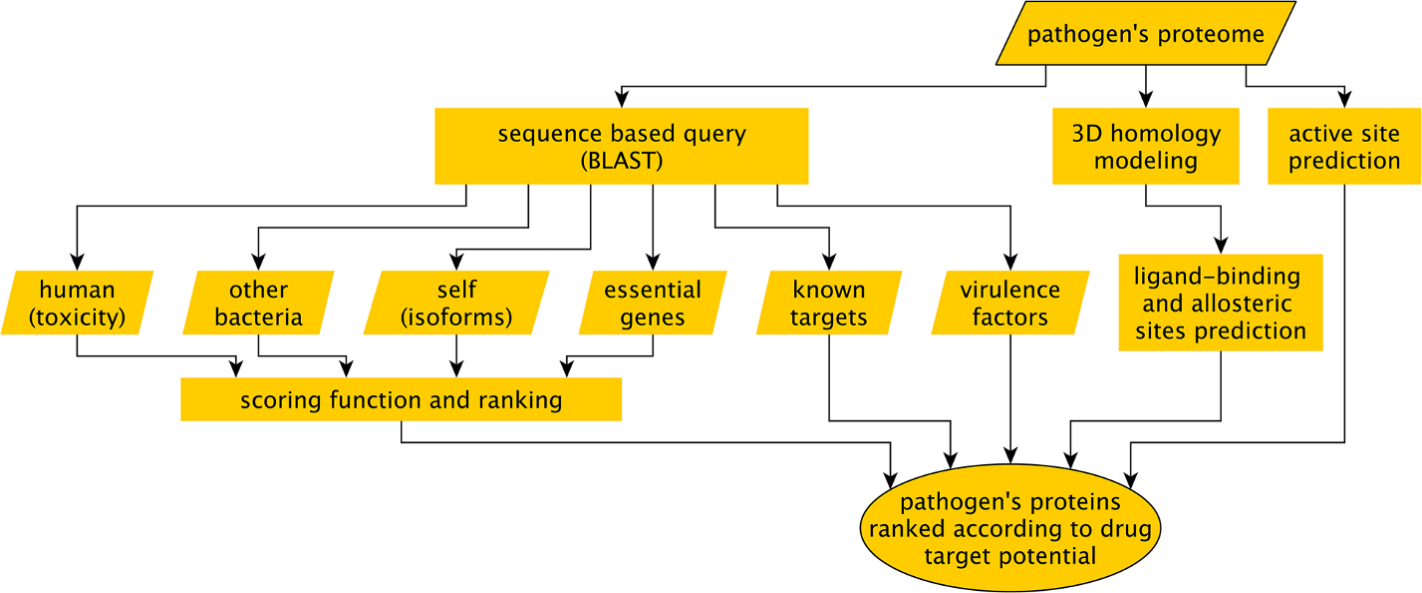
**Pipeline flowchart.** Each proteome of interest was subjected to the computational pipeline depicted in the flowchart. Each protein sequence is compared using BLASTPGP against: (1) the human proteome to look for similarities that could lead to toxicity, (2) other Gram-negative bacteria to estimate the evolutionary conservation, (3) its own proteome to find isoforms and paralogs that could reduce effectivity and promote resistance, (4) the database of essential genes (DEG). The results of these queries are combined into a scoring function as described in the main text. To integrate further relevant information, protein sequences are also queried against known drug targets (DrugBank) and virulence factors (VFDB). In a complementary manner, active sites are predicted as well and, if proper structural templates are found, the protein structure is modeled by homology. If the structure is modeled successfully, ligand-binding sites are predicted and analyzed as described in the main text. The pipeline’s output is a ranked list of the organism’s proteins based on the comparative genomics analysis (*i.e.* essentiality, evolutionary conservation, toxicity and paralogs scores) with links to external databases (VFDB and DrugBank) and when possible, to the corresponding structural modeling and analysis results.

The pipeline to which each proteome of interest was subjected is illustrated in Figure 1, which summarizes the approach.

### Sequence-based analysis

Currently, the database covers 74 Gram-negative pathogens, including 224 distinct strains. In the case of *Acinetobacter baumannii*, *Escherichia coli*, *Helicobacter pylori*, *Pseudomonas aeruginosa* and *Stenotrophomonas maltophilia*, which are pathogens distinguished by their prevalence in community and/or nosocomial infections and the incidence of drug-resistant isolates [3, 10], we included all available fully sequenced strains (82 strains, which conform a ‘priority set’). As for the rest of the species, we included all strains that were marked as ‘human pathogens’ in HAMAP (142 strains) [22]. This query data set was compared against the human proteome and a reference set of 770 Gram-negative proteomes (494 distinct species), by means of the BLASTPGP program [23] using default parameters. BLASTPGP searches are very fast, however resulting E-values depend on the alignment itself and on other parameters such as the size of the database scanned. We needed unbiased similarity scores between proteins matched during the sequence-based search to keep results valid in case of further increasing the size of the data sets. To attain this objective, we further aligned BLASTPGP matches (E-value <= 0.0001) using the Smith-Waterman algorithm and calculated ‘normalized sequence similarity scores’ (NS). NS values were then used to pre-compute toxicity, presence of isoforms or paralogs and the two conservation scores for each protein in the query data set, as described in further detail in the Methods Section.

### Queries to external databases

Besides comparative analysis, a round of database queries was also performed to integrate additional information. Thus, protein sequences were compared to the list of known drug-targets available at the DrugBank database [14] and sequence-based searches were also performed against virulence factors available at the Virulence Factors DataBase (VFDB) [15]. Out of the total 777,585 proteins in the query data set, 375,016 matched a known target in DrugBank (48%) and 193,135 proteins matched a known virulence factor at VFDB (25%). This information was not incorporated as ranking scores, but it is available through the web-interface described below for researchers to evaluate themselves the relevance of such matches in each particular case.

### Three-dimensional homology modeling

Researchers evaluating prospective drug targets may benefit from the availability of protein structural data. For the organisms in the priority set, we performed a large-scale homology modeling of all protein sequences for which we found valid structural templates as explained in the Methods Section. In total, we generated three-dimensional homology models for 136,141 proteins (covering 47% of the priority set). This number was obtained after discarding models presenting less than 30% sequence identity (target-template) or G-factors below -1.00 [24]. All models were generated by means of the MODELLER program [25] using default parameters.

To save computational power, proteins belonging to other strains were not modeled automatically. However, if the user is interested in obtaining one of such homology models, we have implemented an option at the web-interface for automatic submission of the selected modeling task.

### Active-site prediction

To further add relevant information on putative targets, we applied a sequence-based approach [26] to predict the location of active-site residues. The method is based on comparing query sequences to homologs for which the position of the active site has been annotated. After analyzing the whole query set (777,585 proteins), this procedure predicted the location of active-site residues for 90,482 proteins (11.6%).

Proteins with a predicted active site display a link to the details of the prediction in the web interface described below.

### Pocket analysis

For proteins for which we could build a three-dimensional homology model, we predicted the location of ligand-binding sites on the structure using LIGSITEcs [27]. We further analyzed the ligand-binding sites using two previously developed methodologies which estimate the regulatory potential of particular ligand-binding pockets. When possible, the structural conservation of predicted pockets was measured considering the evolutionary record of the protein family, given that conserved pockets may have a relevant biological role [12]. Furthermore, using Normal Mode Analysis we estimated the effect of ligand binding on overall protein flexibility, a measure which has been used in combination with structural conservation to predict the location of allosteric sites [13]. As described below, the user can visualize the protein structure and predictions online.

### Interface and access to results

Interactive access to results is available through the web-interface at ‘http://bioinf.uab.cat/antibactr’.

This interface allows the user to select the organisms and strain of interest, set custom weights to the different scores and then proceed to calculate the corresponding ranking. If the user wishes to ignore a specific ranking parameter, a weight of 0 (zero) can be applied. The system has been built in such a way that normalization of scores is performed only among the selected set of strains and parameters. To further facilitate the analysis of results, the user may also limit the amount of top-ranked entries that are displayed. Once the ranking procedure is finished (it takes a few seconds), the ranking is printed to the browser. An option is available for downloading the ranking to the local computer in tab-delimited text format, useful for researchers interested in further processing the data. Targets are displayed in ranked order and individual scores are shown for each protein after normalization but prior to weighting. A brief description of the biological function is displayed for each protein but, to facilitate immediate access to full annotation and other relevant data, a link to the related Uniprot entry is provided as well [28]. In cases where the target shows sequence similarity to an already known drug target or virulence factor, the corresponding links are also provided. In addition, specific links with details on predicted active sites and homology models are given. If a homology model is supplied, the user may download model coordinates in PDB format and target-template alignments generated during the modeling process, along with sequence identity, DOPE score and other relevant modeling data [25]. Furthermore, available protein structures can be visualized using *Jmol* (http://www.jmol.org) along with the results of the pocket analysis previously described [13].

### User query sequences

Besides the ranking of complete proteomes, researchers may want to look at the ranking of a few selected proteins of their particular interest. To achieve this functionality, we added the possibility to include the user’s own query sequences in an optional field. These sequences are then compared using BLASTPGP against our query data set (224 strains). Scoring and ranking proceed as normally, but results are then displayed only for significant hits within our data set. Details of this BLASTPGP search are also available to the user.

## Discussion

Large-scale comparison of organisms at the genome level is a technique common to many fields of biology and medicine. In the past years complex approaches involving phylogenetic and metabolic studies have been published [29, 30]. However, comparative genomics initiatives in drug discovery have been criticized for their limited success in finding new active compounds [31, 32]. Yet, comparative genomics and proteomics continue to shed light on the workings of bacterial drug-resistance and virulence [33, 34].

Far from attempting to solve the problem of target identification in one strike, our motivation was to implement a straightforward computational approach that would prove useful as an initial filtering and ranking step, aiding researchers in the quest for novel drug targets.

At the time of this writing no equivalent tool to the one presented here is available, however a few servers provide slightly related functionalities and could be used in a complementary fashion. For example the Prokaryotic-genome Analysis Tool (PGAT), developed by Brittnacher and collaborators is a general comparative genomics tool focused particularly on comparing different strains of the same species [17]. PGAT allows the user to carry out a series of interesting analyses including information on metabolic pathways, but it does not provide specific drug-target related information, unlike the UniDrug-Target (UDT) database which clearly focuses on that aspect [16]. The latter presents candidate targets as proteins which are present in pathogenic bacteria but absent in commensal strains. This is a reasonable approach which in our tool can be achieved by setting a negative value for the strain conservation score and it is also one of the functions available at PGAT. However, given its focus on pathogen-specific proteins UDT’s approach tends to discard evolutionary conserved proteins, such as many well known broad-spectrum targets [35].

The amount of well known and characterized protein drug targets is currently in the order of hundreds [35]. To illustrate the potential of the tool presented here, we provide a few examples of already known antibacterial targets. Certain proteins involved in the replication of DNA are targeted by fluoroquinolones [35, 36]. For example, Ciprofloxacin targets DNA topoisomerase 4 subunit B and DNA gyrase subunit A. Even though resistance to fluoroquinolones has been observed in pathogens with mutations in these proteins, new compounds with antibacterial activity on the resistant strains are being developed by studying these targets [37]. These proteins appear on the top 3% of the full proteome ranking for *Escherichia coli* K12 (positions 68 and 158, respectively) when we build the ranking using default parameters. This is because both proteins are essential, show high levels of evolutionary conservation but low similarity to human proteins (minimal potential toxicity) and present no isoforms or paralogs according to our pipeline parameters.

**Figure 2:**
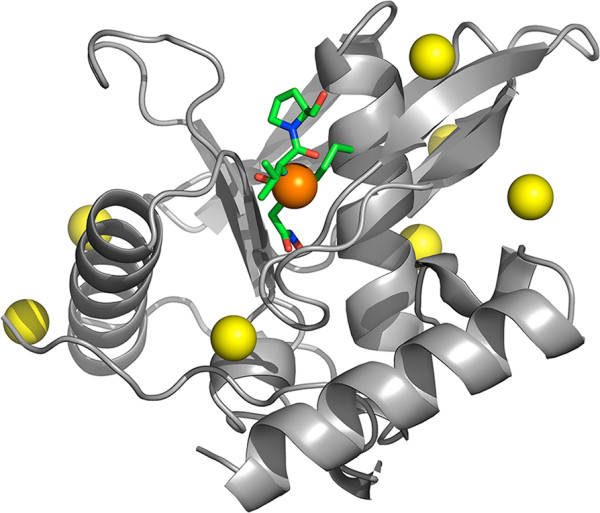
**Peptide deformylase, automatic structural modeling and pocket analysis.** The spheres displayed on the homology model of *Pseudomonas aeruginosa* peptide deformylase [Swiss-Prot:Q9I7A8] (based on template [PDB:1N5N]) represent putative ligand-binding sites as predicted by the automatic pocket analysis. The orange sphere marks the only cavity predicted to significantly affect overall protein flexibility. To illustrate the relevance of this prediction, we show the location of the antibiotic ligand (in ‘sticks’ representation) after superimposing the homology model to the known structure of the antibiotic-bound protein [PDB:1LRY] (RMSD 0.5 Å). The position of antibiotic Actinonin matches precisely the cavity marked by the procedure. The same cavity is also estimated to be very well conserved at the structural level (100% presence in the protein family).

Beyond filtering and ranking targets, researchers can also gain insight into potential targets through the structural analysis methods we have implemented into the tool. For example, peptide deformylase [Swiss-Prot:Q9I7A8] from *Pseudomonas aeruginosa* is an essential protein targeted by the antibiotic Actinonin [38]. This protein is ranked at position 81 among the complete *Pseudomonas aeruginosa* proteome (top 2%) when using default parameters. Our pipeline automatically builds three-dimensional homology models when possible, it then predicts putative ligand-binding sites and evaluates their potential to regulate protein activity as described previously [13]. In this case, the structural analysis (which is pre-calculated and available through the web-interface) predicts one of the putative ligand-binding sites to significantly affect protein flexibility as shown in Figure 2. When we superimpose the automatically generated homology model with the known structure of the protein bound to the antibiotic ([PDB:1LRY] RMSD 0.5) the position of the cavity predicted to be significant matches precisely the location of the antibiotic molecule. This cavity is also considered relevant from an evolutionary perspective, as it shows 100% of structural conservation within its domain family according to the corresponding automatic analysis [12]. The structural conservation of this pocket was to be expected, since it is the protein’s active site. Briefly, this case illustrates how in the situation of a poorly characterized protein, our automatic structural analysis may pinpoint not only the potential of the protein as a drug target, but the precise location of the drug-binding pocket.

**Figure 3:**
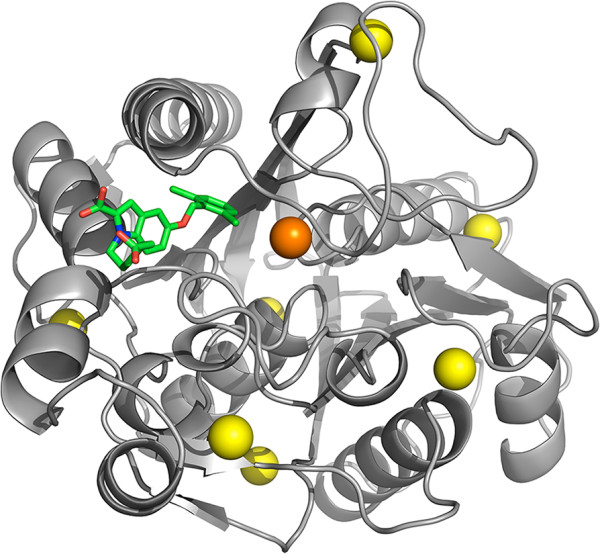
**Automatic structural modeling and pocket analysis of 3-oxoacyl-[acyl-carrier-protein] synthase 3.** The spheres displayed on *Escherichia coli*’s 3-oxoacyl-[acyl-carrier-protein] synthase 3 [Swiss-Prot:P0A6R0] homology model (based on template [PDB:1UB7]) represent putative ligand-binding sites as predicted by the automatic pocket analysis. The orange sphere marks the only cavity predicted to significantly affect overall protein flexibility. This predicted position occupies the same cavity and is very close (4.1 Å) to the known inhibitor molecule (in ‘sticks’ representation) after superimposing the homology model to the known structure of the inhibitor-bound protein [PDB:1MZS] (RMSD 1.1 Å). This shows how automatic homology modeling and pocket analysis are combined to correctly identify a regulatory cavity.

Another interesting example is 3-oxoacyl-[acyl-carrier-protein] synthase III, which is involved in fatty-acid synthesis. This protein is targeted by Cerulenin, with antifungal effects, and it is a potential antibacterial target as well [39–41]. It is ranked by our pipeline at position 49 (top 2%) among the full proteome of *Escherichia coli* K12 because it is essential and well conserved, showing in principle no toxicity (similarity to human proteins). Furthermore, structural analysis reveals one single pocket that could affect the protein’s function by perturbing its overall flexibility, as shown in Figure 3. When we evaluate the structural prediction performed on the homology model by superimposing the known structure of the inhibitor-bound protein, we observe that in this case the match of the cavity’s geometric center is not as precise as in the previous example of peptide deformylase. Nevertheless, visual inspection shows that the large cavity indicated by the pocket structural analysis is indeed occupied by the inhibitor, so that the automatic procedure would again be pointing the researcher in the right direction, even if no inhibition information would have been available *a priori*.

**Figure 4:**
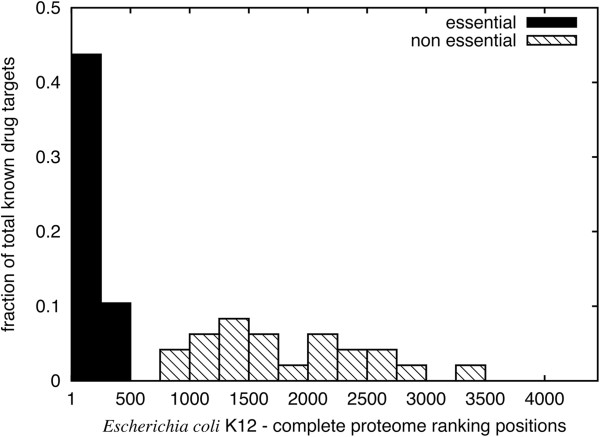
**Ranks distribution for known antibacterial targets.** The histogram displays the distribution of ranks for 48 DrugBank known targets in *Escherichia coli* strain K12 within the complete proteome ranking using default parameters. Target proteins known to be essential according to DEG appear at higher ranking positions (solid black), while non-essential proteins appear further down in the ranking (hatch pattern).

Of course, multiple other factors beyond the reach of a mere computational approach participate in defining a protein as a good antibacterial target. Indeed the final outcome of clinical trials can hardly be predicted [31]. However, we considered relevant to infer how well the pipeline presented in this work ranks already known targets. We gathered all known targets of ‘approved’ drugs, as annotated in DrugBank [14], that belonged to any of the strains analyzed in this work. We found a total of 57 proteins identified through their Uniprot ID [28]. Of those 57, a majority (48) belong to *Escherichia coli* K12. When we proceed to rank this organism’s proteome, half of the known drug targets appear at the top 10% of the ranking. The distribution of the 48 known targets in the ranking of *Escherichia coli* K12 is displayed in Figure 4. It is interesting to note that the first half or top 10% correspond to essential proteins. Since essentiality is a binary score (*i.e.* genes may be essential or not), it divides the ranking in two sections as can be seen in the histogram (Figure 4). This illustrates the difficulty of *a priori* ranking antibacterial targets, since even though essentiality is considered a very desirable property for any candidate target [32, 42, 43], it only represents half of the known targets in this organism. Moreover, assessing gene essentiality is not a trivial task, given that *in vitro* results do not always correlate with gene essentiality determined *in vivo*[43].

Because the function that would unequivocally assign target scores to proteins is highly complex and full of unknowns, the pipeline presented here has been developed with the sole aim to assist the selection of prospective candidates, it is not meant to provide a final or complete list of antibacterial targets. Very often, it will not be used as a ranking tool but to retrieve target-relevant information for a specific protein and evaluate its pros and cons with respect to other potential candidates. The versatility of the tool, with dynamic features such as the assignation of relative weights at the user’s criterion, and the availability of original information, such as predicted, functionally relevant ligand-binding sites, may prove valuable arguments for the microbiologist or medicinal chemist researching on new antibacterial targets.

## Conclusions

We have developed a database and web-based tool for the ranking of proteins from a set of user-selected bacterial proteomes according to a series of antibacterial-drug-target-like properties. Specifically, the tool evaluates (1) the presence of the protein in other Gram-negative species (currently 494) to assess conservation at this level and, by extension, the potential spectrum of a drug targetting this protein, (2) the identification of the protein as essential for bacterial survival or growth, (3) the presence of homologs in the human genome to assess potential toxicity or side effects of a drug targetting this protein and (4) the presence of paralogs or isoforms of the protein in the same proteome, which could facilitate the development of resistance and reduce effectivity. The user may choose the weight of each of these four properties in the ranking, including negative weights (e.g. for homology to human and presence of isoforms or paralogs). When available, the ranked proteins incorporate, as additional information, any found matches to proteins in the DrugBank (i.e. known drug targets) and the Virulence Factors Database (VFDB), as well as a model of the protein’s three-dimensional structure and an analysis of active and regulatory sites to assess druggability of the potential target. This is currently the single resource combining sequence-based and structural information for the identification of potential antibacterial-drug targets in multiple genomes.

Currently, the database covers 74 Gram-negative pathogens, including 224 distinct strains. The number of strains is particularly extended for *Acinetobacter baumannii*, *Escherichia coli*, *Helicobacter pylori*, *Pseudomonas aeruginosa* and *Stenotrophomonas maltophilia* (82 strains in total), which are found among a group of Gram-negative pathogens distinguished by prevalence in either community or nosocomial infections and incidence of drug-resistant isolates. Additional strains and species will be added to the database in subsequent updates.

## Methods

### Normalized sequence-similarity score (NS)

We used the BLASTPGP program [23] with default parameters to scan complete proteomes. Since BLASTPGP E-values may vary depending on the size of the queried database, we aligned all matched pairs and calculated their Smith-Waterman similarity score [44]. We ignored alignments with scores lower than 100, as previously described [45].

Given that the Smith-Waterman similarity score is related to the size of the alignment, we divided the score by the length of the alignment to obtain a normalized sequence-similarity score (NS). The Smith-Waterman algorithm computes an optimal local alignment, meaning that the NS measure of similarity between two proteins is equivalent to the similarity between their most closely related pair of domains or regions.

### Essentiality

Experimental information regarding gene essentiality is available for a few organisms at the database of essential genes (DEG) [20]. If a particular strain was not available at DEG, we mapped query proteins to essential genes by using BLASTPGP. For each annotated essential gene in a related strain, we scanned the proteome of interest and marked the best hit as an essential gene. Only E-values of 1e-10 or better were considered acceptable for this task. At the time of this writing, we were only able to gather large-scale essential gene information for: *Acinetobacter baumannii*, *Escherichia coli*, *Helicobacter pylori*, *Pseudomonas aeruginosa* and *Vibriocholerae*.

### Toxicity

An antibacterial drug acting on protein targets which are similar to human proteins may also bind these causing adverse effects and/or toxicity. We estimated the potential toxicity of each putative target proportional to the largest NS value obtained after pairwise alignment against the whole human proteome.

### Isoforms and paralogs

If a given drug target presents multiple isoforms or paralogs (‘variants’), the pathogen may readily develop resistance by functional substitution mechanisms. It is also possible that the drug may bind both the target and its variants, thus decreasing the antibiotic effect. To assess this parameter for each potential drug target, we counted the amount of variants present in the same proteome. We considered as variants of a protein all similar proteins with a NS value equal or larger than 2.

### Evolutionary conservation among Gram-negative organisms

We defined a score to estimate the evolutionary conservation of potential targets across Gram-negative (GN) organisms as follows:
1

Where *GNC*_*p*_ is the Gram-negative conservation score for protein *p*, computed by adding the highest NS value (max (*NS*_*p*_)) obtained against each of the different GN species (*i*) in the data set, with *n* being the total number of GN species.

### Conservation among strains

We estimated the evolutionary conservation of proteins among different strains of the same species using the following score:
2

where *SC*_*p*_ is the strain conservation score for protein *p*, computed by adding the highest NS value (max (*NS*_*p*_)) obtained against each other strain (*j*) of the selected species in the data set, with *m* the total number of distinct strains of the particular species.

### Scoring function and ranking of potential drug targets

Each of the different scores is normalized by the largest value obtained across the selected organisms. Normalized values are then multiplied by 100 to obtain percentages, *i.e.* final scores range between 0 and 100.

Each independent score has an associated weight, which can be negative or positive. These weighting values can be set by the user. However, default values are provided as follows. *A priori* negative features of a putative target (*i.e.* Toxicity and Paralogs) are given a default weight of -1, while positive features (*e.g.* Evolutionary conservation, Essentiality) have a corresponding default weight of 1.

For each protein in the selected data set, normalized scores are multiplied by their respective weights. The final score for each protein is obtained by summing up all weighted scores. Finally, all proteins in the selected data set are ranked according to their final score in terms of drug-target potential.

### Comparative-genomics reference data set

Sequence data on Gram-negative (GN) organisms was gathered for a total of 770 fully sequenced GN proteomes covering 494 distinct species. GN bacteria species were identified at ‘http://www.bacterialphylogeny.com/bacteria.html’ and fully sequenced bacterial proteomes at ‘http://www.uniprot.org/taxonomy’ using the query string: ‘*bacteria AND complete:yes*’. A total of 770 bacterial strains were common to both listings. We downloaded sequence data from ‘ftp://ftp.expasy.org/databases/complete_proteomes/fasta/bacteria/’.

### Known drug targets and virulence factors

Each proteome of interest was compared by means of the BLASTPGP program [23], with default parameters, against known drug targets available at DrugBank [14] and virulence factors available at VFDB [15]. Proteins showing a match with a BLASTPGP E-value <= 1e-2 display a link to the related hits in the output table.

### Three-dimensional homology models

An automated homology-modeling pipeline was implemented based on the program MODELLER v9.5 [25]. Briefly, for a given protein sequence the system scans a database of structural templates. It then proceeds to generate homology models using the best possible set of diverse templates that display at least 30% sequence identity. Finally, the best resulting models are selected using a combination of DOPE and GA341 scores [25].
